# Nocturnal short-term heart rate variability reflects impaired daytime vigilance better than overnight heart rate variability in suspected obstructive sleep apnea patients

**DOI:** 10.1093/sleep/zsae282

**Published:** 2024-12-13

**Authors:** Salla Hietakoste, Tuomas Karhu, Carolina Lombardi, Pablo Armañac-Julián, Raquel Bailón, Brett Duce, Saara Sillanmäki, Juha Töyräs, Timo Leppänen, Sami Myllymaa, Samu Kainulainen

**Affiliations:** Department of Technical Physics, University of Eastern Finland, Kuopio, Finland; Diagnostic Imaging Center, Kuopio University Hospital, Kuopio, Finland; Department of Technical Physics, University of Eastern Finland, Kuopio, Finland; Diagnostic Imaging Center, Kuopio University Hospital, Kuopio, Finland; Department of Cardiology, Neural and Metabolic Sciences, Sleep Disorders Center, San Luca Hospital, IRCCS Istituto Auxologico Italiano, Milan, Italy; School of Medicine and Surgery, University of Milano-Bicocca, Milan, Italy; Centro de Investigación Biomédica en Red en Bioingeniería, Biomateriales y Nanomedicina (CIBER-BBN), Instituto de Salud Carlos III, Madrid, Spain; Biomedical Signal Interpretation and Computational Simulation (BSICoS) Group, Aragón Institute of Engineering Research (I3A), University of Zaragoza, Zaragoza, Spain; Centro de Investigación Biomédica en Red en Bioingeniería, Biomateriales y Nanomedicina (CIBER-BBN), Instituto de Salud Carlos III, Madrid, Spain; Biomedical Signal Interpretation and Computational Simulation (BSICoS) Group, Aragón Institute of Engineering Research (I3A), University of Zaragoza, Zaragoza, Spain; Department of Respiratory and Sleep Medicine, Sleep Disorders Centre, Princess Alexandra Hospital, Brisbane, Australia; Institute for Health and Biomedical Innovation, Queensland University of Technology, Brisbane, Australia; Diagnostic Imaging Center, Kuopio University Hospital, Kuopio, Finland; Institute of Clinical Medicine, University of Eastern Finland, Kuopio, Finland; Department of Technical Physics, University of Eastern Finland, Kuopio, Finland; Science Service Center, Kuopio University Hospital, Kuopio, Finland; School of Electrical Engineering and Computer Science, University of Queensland, Brisbane, Australia; Department of Technical Physics, University of Eastern Finland, Kuopio, Finland; Diagnostic Imaging Center, Kuopio University Hospital, Kuopio, Finland; School of Electrical Engineering and Computer Science, University of Queensland, Brisbane, Australia; Department of Technical Physics, University of Eastern Finland, Kuopio, Finland; Diagnostic Imaging Center, Kuopio University Hospital, Kuopio, Finland; Department of Technical Physics, University of Eastern Finland, Kuopio, Finland; Diagnostic Imaging Center, Kuopio University Hospital, Kuopio, Finland

**Keywords:** obstructive sleep apnea, heart rate variability, hypoxic load, cardiorespiratory coupling, psychomotor vigilance task

## Abstract

In obstructive sleep apnea (OSA), heart rate variability (HRV) decreases and performance in psychomotor vigilance task (PVT) worsens with more severe hypoxic load. Nevertheless, the association between HRV and PVT performance is poorly understood. Thus, we hypothesize that nocturnal short-term HRV is better related to daytime psychomotor vigilance compared with overnight HRV. To investigate this hypothesis, we retrospectively analyzed the electrocardiograms from polysomnographies of 546 consecutive patients with suspected OSA. We determined overnight HRV and short-term HRV in nonoverlapping 5-min segments and performed stepwise linear regression analyses to associate HRV with the median reaction time (RT) in the PVT. The short-term decrease in the median interval between two successive normal R peaks (NN interval), root mean square of successive NNs, and normalized high-frequency band power were all significant (*p* < 0.001) indicators of longer median RTs. However, the overnight HRV parameters did not indicate worsening median RT. Instead, increased hypoxic load and N3 duration were associated with longer median RT in men but not in women. The association of HRV and cardiorespiratory coupling with PVT performance was generally weak. Nocturnal short-term HRV evaluation reflected a state of vigilance better than the average overnight HRV. Thus, the overnight HRV analysis might not be optimal for patients with OSA. Utilizing the HRV analysis in a time-series manner and combined with the hypoxic load and sleep stages could bring new aspects to the health assessment of patients with OSA.

## Graphical Abstract

**Graphical Abstract:**
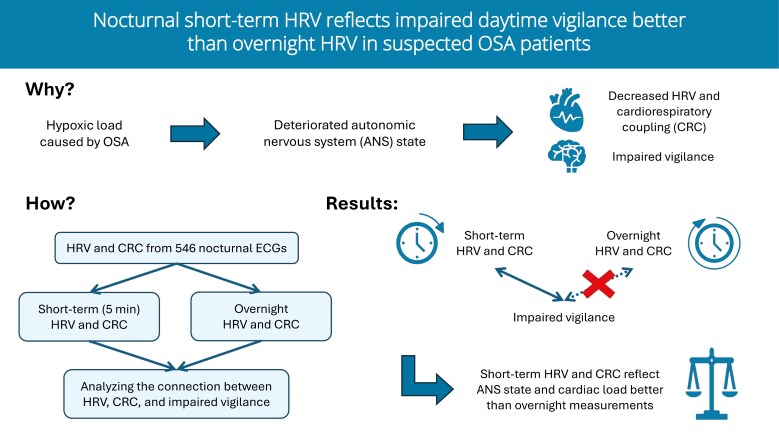

**Statement of Significance**

Long-term heart rate variability (HRV) is considered as a biomarker of health. However, nocturnal short-term HRV was linked to impaired daytime vigilance in our study whereas long-term overnight HRV was not. These novel findings emphasize that by averaging HRV overnight, we lose a marked amount of information on vagal modulation and physiological responses on unstable breathing patterns in patients with obstructive sleep apnea (OSA). Thus, the overnight HRV analysis might not be optimal for patients with OSA. Patients with OSA could benefit from more thorough HRV analysis combined with an assessment of hypoxic load severity as short-term HRV analyses enable the detection of subtle variations in autonomic cardiac regulation and early signs of sleep-related health risks.

## Introduction

Obstructive sleep apnea (OSA) is a growing global health problem that affects approximately 1 billion people [1, 2]. In OSA, fragmented sleep due to nocturnal respiratory events (i.e. apneas and hypopneas) gradually leads to excessive daytime sleepiness (EDS), impaired daytime vigilance, and decreased neurocognitive performance with difficulties sustaining attention [3, 4]. Therefore, OSA puts pressure on health care systems as these patients have a higher risk for other comorbidities as well as for work and traffic accidents [5, 6].

The psychomotor vigilance task (PVT), which measures reaction times (RTs) to repeated visual stimuli, is a quick and objective way to assess an individual’s ability to sustain attention [7, 8]. Despite the PVT producing sensitive markers for impaired vigilance [9, 10], it is not a routine test in the current clinical OSA diagnostics although people with OSA exhibit lower performance in PVT compared with healthy individuals [3, 11]. Previous studies [4, 11], however, demonstrate a poor connection between PVT outcomes and conventional parameters measuring OSA severity (i.e. the apnea–hypopnea index, AHI), its worsening, and the degree of sleep fragmentation (i.e. the arousal index). Instead, impaired vigilance and EDS are better associated with nocturnal hypoxic load and its severity [11–16].

The hypoxic load associated with OSA also has negative effects on cardiorespiratory health. The autonomic nervous system (ANS) is influenced by hypoxic load, which causes a shift in the sympathovagal balance toward sympathetic dominance [6]. These alterations can be assessed noninvasively using heart rate variability (HRV) [17]. Elevated sympathetic nervous system (SNS) activity increases heart rate (HR), decreases long-term HRV indicating poor health, and causes oxidative stress [6, 17]. The severity of hypoxic load is directly related to the level of SNS activation [18] and the degree of acute cardiorespiratory coupling (CRC) impairment [19]. A high level of CRC in the high-frequency (HF) band (0.15–0.40 Hz) is considered a dependable biomarker for stable sleep and breathing in healthy adults [20–22].

Previously, HRV has been used to detect impaired vigilance in sleep-deprived patients [23, 24], but conflicting findings have emerged when investigating the relationship between HRV and EDS in patients with OSA [25]. Thus, the connection between HRV and diminished alertness in OSA patients remains unclear. Given that hypoxic load impacts both HRV and PVT performance, HRV characteristics could be utilized to assess the vigilance of patients with OSA. HRV can be readily computed as electrocardiography (ECG) is routinely recorded in polysomnography (PSG) and it could be easily attached to home sleep apnea tests. Thus, our objective was to investigate whether HRV varies between patients with OSA who perform well and those who perform poorly in the PVT. We hypothesized that patients who experience a decrease in HRV and impaired CRC during sleep exhibit longer RTs and a higher number of lapses in the PVT.

## Methods

### Dataset

The initial clinical population comprised 901 consecutive patients undergoing type I PSG and PVT. The patients were referred for sleep study due to an OSA suspicion in the Sleep Disorders Center of Princess Alexandra Hospital (Brisbane, Australia) between 2015 and 2017. The PSGs were recorded using the Compumedics Grael acquisition system (Compumedics, Abbotsford, Australia). Experienced sleep technicians manually scored the PSG recordings in compliance with the prevalent American Academy of Sleep Medicine (AASM) 2012 scoring criteria using the 3% desaturation rule for hypopneas [26]. The retrospective data collection and reuse were approved by the Metro South Human Research Ethics Committee (HREC/16/QPAH/021 and LNR/2019/QMS/54313).

**Figure 1.**
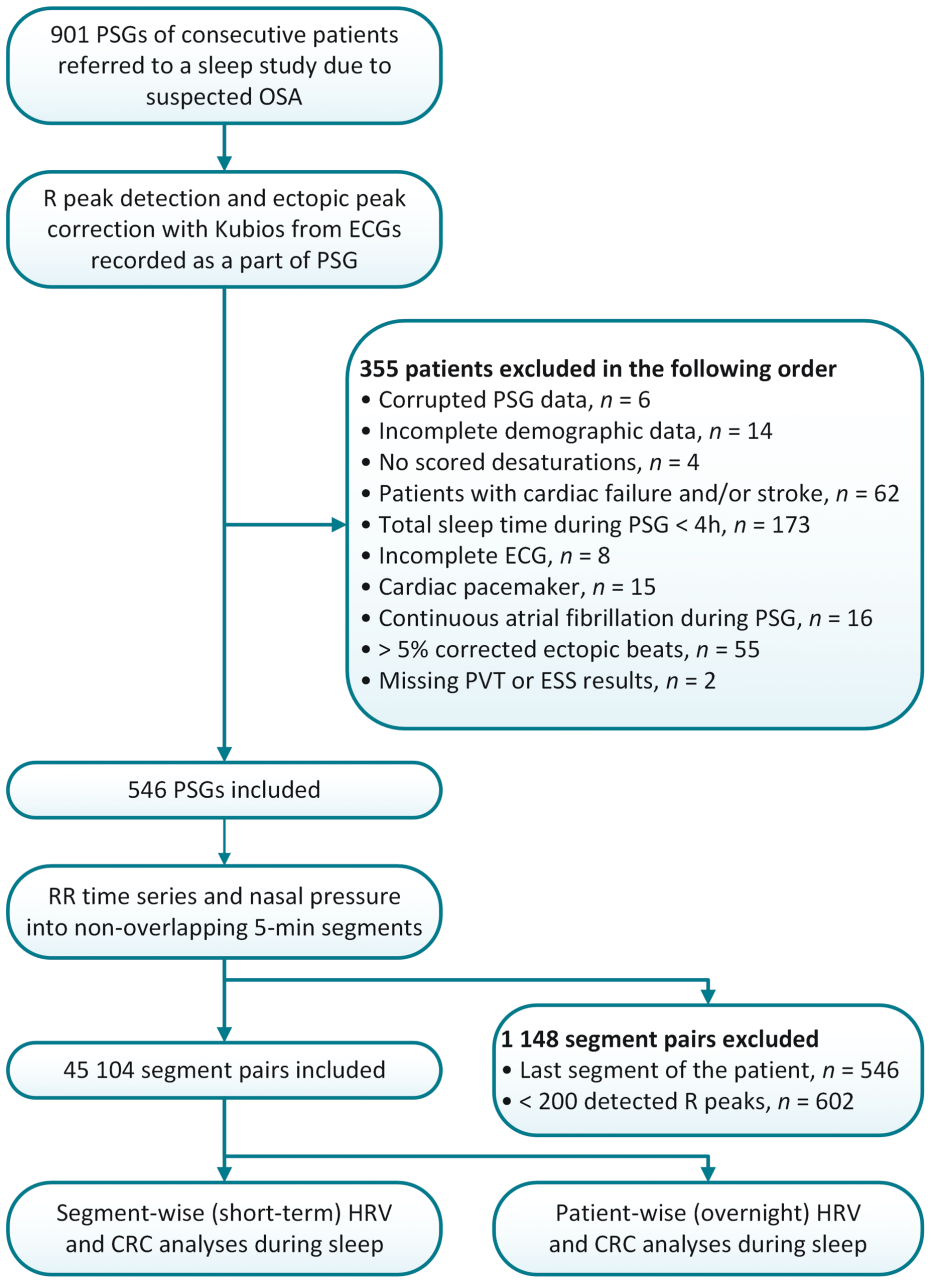
Inclusion and exclusion criteria for patients and 5-min segment pairs of R peak time series and nasal pressure selection. Abbreviations: CRC, cardiorespiratory coupling; ECG, electrocardiogram; ESS, Epworth sleepiness scale, HRV, heart rate variability; Kubios, Kubios Premium 3.4.1 software (Kubios Oy, Kuopio, Finland); OSA, obstructive sleep apnea; PSG, polysomnography; PVT, psychomotor vigilance task.

As HRV parameters are reliable markers of the ANS state when the heart beats in sinus rhythm, we excluded patients with cardiac conditions potentially affecting the heart functioning and sinus rhythm (total of *n* = 148, Figure 1). We also excluded patients whose PSG or ECG recording failed or whose demographic data were incomplete (total of *n* = 32), and patients who slept less than 4 h during the PSG (*n* = 173) to ensure having high-quality data and the most descriptive population of OSA patients as possible. After patient exclusion, we included a total of 546 (52.7% of men) patients in this study (Table 1) [27].

**Table 1.** Characteristics of the study population

	All	Men	Women
Number of patients, *n* (%)	546 (100%)	288 (52.7%)	258 (47.3%)
Age [y]	51.8 (41.3–62.0)	52.2 (41.4–62.5)	51.3 (41.0–60.3)
BMI [kg/m^2^]	34.0 (28.7–39.8)	32.5 (27.8–38.3)	36.1 (29.9–43.3)
AHI [events/h]	13.0 (5.7–25.5)	17.7 (8.4–32.9)	9.2 (3.8–17.2)
No OSA, *n* (%)	120 (22.0%)	38 (13.2%)	82 (31.8%)
Mild OSA, *n* (%)	180 (33.0%)	87 (30.2%)	93 (36.0%)
Moderate OSA, *n* (%)	135 (24.7%)	87 (30.2%)	48 (18.6%)
Severe OSA, *n* (%)	111 (20.3%)	76 (26.4%)	35 (13.6%)
ODI_3%_ [events/h]	12.3 (3.8–31.1)	18.5 (6.1–40.2)	7.1 (2.3–20.8)
Total sleep time [min]	333.8 (289.5–374.5)	326.0 (282.8–367.3)	340.8 (304.0–383.5)
PVT			
Median RT [ms]	376 (341–437)	361 (330–407)	398 (360–469)
Lapses [*n*]	12.5 (5–34)	9 (4–20)	17 (8–43)
ESS score	10 (6–14)	10 (6–14)	11 (6–15)
Comorbidities, *n* (%)			
Atrial arrhythmia^*^	31 (5.7%)	22 (7.6%)	9 (3.5%)
COPD	42 (7.7%)	23 (8.0%)	19 (7.4%)
T2DM	89 (16.3%)	51 (17.7%)	38 (14.7%)
Hypothyroidism	55 (10.1%)	17 (5.9%)	38 (14.7%)
Hypertension	199 (36.4%)	103 (35.8%)	96 (37.2%)

Values are presented as medians (interquartile range) for continuous variables and as counts (percentages) for discrete variables. The severity of OSA is defined according to AASM guidelines [27]: No OSA = AHI < 5, Mild OSA = 5 ≤ AHI < 15, Moderate OSA = 15 ≤ AHI < 30, Severe OSA = AHI ≥ 30. Abbreviations: AHI, apnea-hypopnea index; BMI, body mass index; COPD, chronic obstructive pulmonary disease; ESS, Epworth sleepiness scale; ODI_3%_, oxygen desaturation index using the 3% desaturation rule for hypopneas; PVT, psychomotor vigilance task; RT, reaction time; T2DM, type II diabetes mellitus.

^*^Patients have a history of arrhythmias but sinus rhythm during PSG.

The patients completed the PVT between 7 and 9 pm on the evening before the PSG. The PVTs were conducted with the 10-min protocol for the Psychology Experiment Building Language (PEBL) program [28]: 121 visual stimuli appeared to each patient occurring every 3.4–12.4 s (each interstimulus interval included a 2-s feedback on response time followed by a 0.4-s fixation period). The patients were instructed to respond to visual stimuli as fast as possible by pressing the button on an external keyboard. The RTs were recorded and from them, the number of lapses (the number of responses with an RT of >500 ms) was calculated.

### Data preparation

We detected the R peaks for the HRV analysis from the ECGs (sampling frequency of 256 Hz, modified lead II [26]) with the Kubios Premium 3.4.1 software (Kubios Oy, Kuopio, Finland) with the default settings [29]. The Kubios utilizes the Pan–Tompkins method [30] in the R peak detection and automatically corrects the artifacts due to ectopic beats and missed peak detections by replacing them using cubic spline interpolation. Excessive ectopic beats interfere with the HRV analysis; therefore, we excluded patients with >5% of corrected peaks in their R peak time series (Figure 1).

We extracted each patient’s R peak time series during sleep and divided them into nonoverlapping 5-min segments. We divided the nasal pressure signal, recorded with a sampling frequency of 128 Hz, into corresponding segments. The last segment pair of each patient was excluded due to most likely being shorter than 5 min. For each 5-min segment pair, the severity of the hypoxic load was described using the desaturation severity (DesSev) parameter [31]. The DesSev parameter measures the degree of the nocturnal hypoxic load by considering the depth and duration of each desaturation event as the integrated area under the blood oxygen saturation curve. It was calculated by normalizing the sum of individual desaturation areas in the segment with the duration of the segment (i.e. 5 min). In this study, the DesSev included the recovery area back to the baseline as the desaturations were scored from baseline to baseline. In addition to the segment-wise DesSev, we computed the overnight DesSev for each patient by normalizing the sum of all desaturation areas during sleep by the total sleep time.

### HRV and spectral coherence

**Figure 2.**
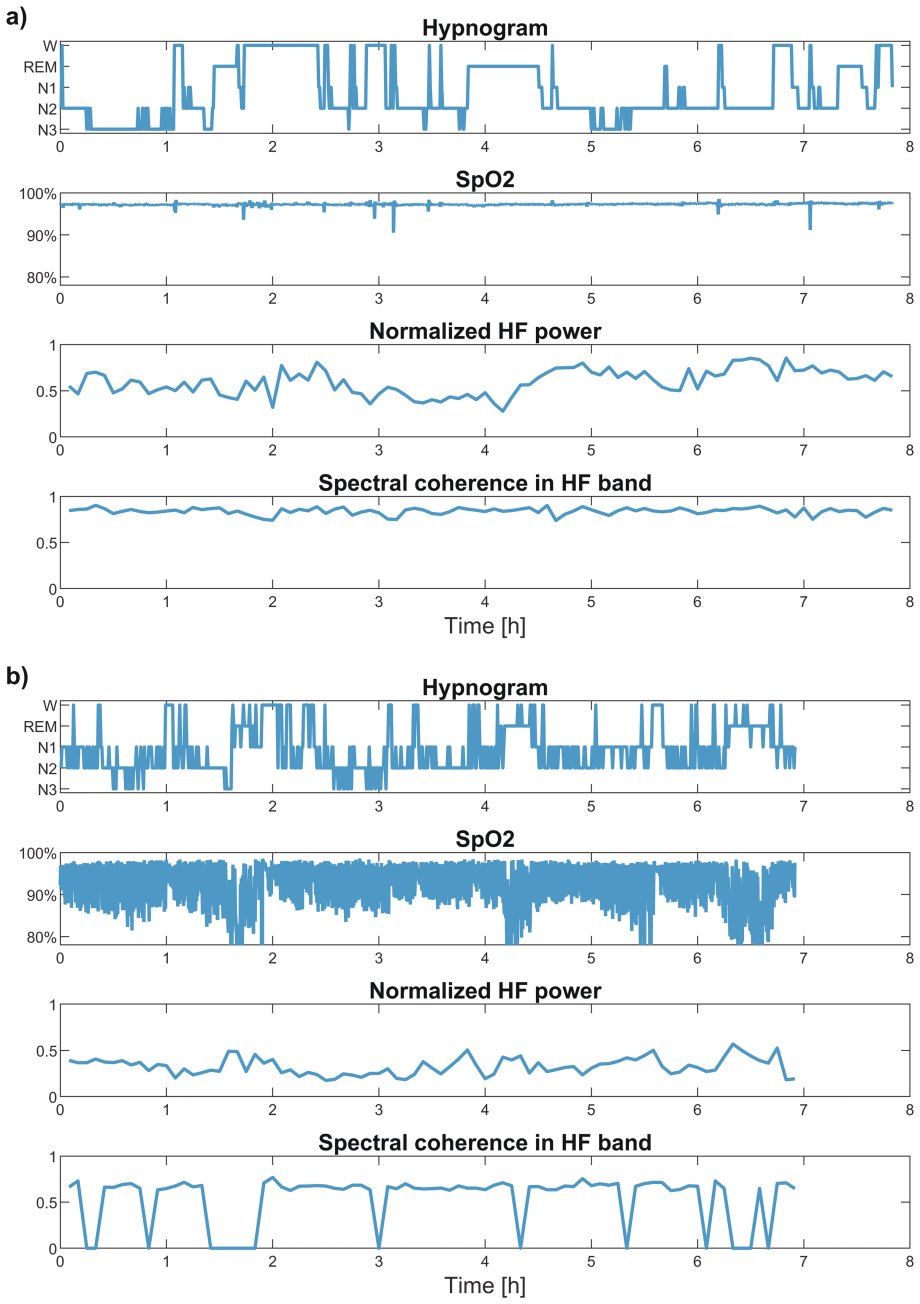
An illustration of heart rate variability (HRV) and cardiorespiratory coupling (CRC) of a patient (a) without sleep apnea (apnea-hypopnea index (AHI) = 0.4 events/h) accompanied by low nocturnal hypoxic load (Desaturation severity (DesSev) = 0.008%) and (b) with severe sleep apnea (AHI = 81.2 events/h) accompanied by severe hypoxic load (DesSev = 5.022%). Intermittent hypoxia over-activates the sympathetitc nervous system and induces arousals from sleep. Together they further lead to fragmented sleep, lower normalized high-frequency (HF) power (decreased HRV), and weakened spectral coherence in the HF band (consistently impaired CRC). The level of significant CRC was based on surrogate data analysis [34]: it is determined only if the coupling level exceeded the threshold (ρ = 0.6241) for significant coupling. Otherwise, the coupling was marked as zero.

We separately computed the short-term (5 min) and long-term (overnight) HRV for men and women to measure their ANS states. For short-term HRV, we calculated the interbeat intervals (interval between two successive normal R peaks, NN interval) from the 5-min R peak time series. We excluded the segments containing <200 R peaks, that is, with an average HR of <40 bpm (Figure 1). In the frequency-domain HRV, we resampled the 5-min NN interval series by cubic spline interpolation to 4 Hz and detrended the signals using the Smoothness priors method [32]. We estimated the power spectral density (PSD) of each 5-min NN time series using Welch’s method (eight windows, 50% overlap, and the Hamming window) and computed the frequency-domain HRV parameters from the PSDs [17, 33]. The parameters comprised the HF band (0.15–0.4 Hz) power, the low-frequency (LF) band (0.04–0.15 Hz) power, the ratio of the LF and HF band powers (LF/HF ratio) as well as the normalized powers for the HF and LF bands (HF_NU_ = HF/(HF + LF) and LF_NU_ = LF/(HF + LF), respectively) [17]. The time-domain HRV parameters were computed from the 5-min NN time series and comprised the average NN interval, the standard deviation of NN intervals (SDNN), the root mean square of successive differences (RMSSD), and the proportion of the NN intervals differing by more than 50 ms from each other (pNN50) [17, 33]. For overnight HRV, the frequency-domain parameters were determined as a mean of the parameter values of the 5-min segments separately for each patient (Figure 2) [33]. For the overnight time-domain HRV parameters, however, we determined the complete NN time series from sleep onset to offset and computed the parameters from that NN series [33].

Next, we computed the degree of short-term CRC as the spectral coherence between the NN time series and corresponding nasal pressure segments. Before the coherence analysis, we downsampled the nasal pressure from 128 to 4 Hz to correspond to the sample rate of the resampled NN time series. We applied Welch’s method (eight windows, 50% overlap, and the Hamming window) to compute the magnitude-squared coherence function on HF and LF power bands (HFc and LFc, respectively). The coherence was computed only if the NN intervals and nasal pressure were linearly related within each power band and the value of spectral coherence exceeded the threshold value for a significant coupling. Otherwise, the coupling was marked as zero. We determined the level of a significant coupling in a 5-min segment (ρ = 0.6241) based on a surrogate data analysis [34]. We accepted an α = 1% risk of obtaining a result indicating a significant coupling between the NN intervals and nasal pressure even though a real coupling did not exist. We have described the threshold value determination in detail in our previous study [19]. In addition to the short-term CRC, we computed the overnight CRC individually for the patients by averaging all their CRCs within the 5-min segments (Figure 2).

### Covariate-adjusted stepwise regression analysis

We performed covariate-adjusted stepwise regression analysis separately for the 5-min segments (*n* = 45 104) and at the patient level (overnight) to consider the effects of sleep stages and comorbidities on the PVT results (median RT and number of lapses) and subjective sleepiness based on the Epworth Sleepiness Scale (ESS). We studied men and women separately. In stepwise regression models, we used the largest set of terms in a linear fit with the Akaike information criterion. We considered demographic factors (i.e. age and the body mass index), HRV, CRC, and sleep stages in minutes as continuous adjusting variates in all our regression models. Comorbidities (chronic obstructive pulmonary disease (COPD), type II diabetes mellitus (T2DM), and hypertension) were considered as the binary adjusting variates. In the segment-wise (short-term) regression models, each segment was associated with the demographic factors, comorbidities, PVT results, and ESS score of the patient from whom the segment was extracted, that is the adjusting data were duplicated for each segment. All HRV parameters were separated into time- and frequency-domain models. We set the limit for the statistical significance to *p* < 0.05 in overnight regression models and to *p* < 0.001 in short-term regression models due to larger sample sizes.

### Patient-by-patient comparison and statistical analysis

We divided the patients into four equal-sized groups (quartiles, *Q*_1_–*Q*_4_) based on their DesSev and HF_NU_. In those quartiles, we calculated the median overnight HRV, CRC, and PVT results to study the effects of overnight DesSev and HRV on daytime vigilance. In addition, we performed a similar comparison between quartiles divided based on the median RT in the PVT to determine the effect of impaired vigilance on HRV. The statistical significance of the difference between quartiles was assessed with the Mann–Whitney *U*-test. Due to the multiple comparisons between the quartiles, we performed the Bonferroni correction and set the limit for the statistical significance to *p* < 0.008. All signal, statistical, and regression analyses were performed with MATLAB R2022b (MathWorks Inc, MA).

## Results

### Effect of HRV and sleep on vigilance

In general, the association of HRV with impaired vigilance was weak in overnight and short-term regression models. Short-term changes, however, were more strongly linked to impaired vigilance.

In overnight models for the median RT in the PVT, we observed that neither HRV parameters nor comorbidities were selected into stepwise regression models and thus, were not associated with the median RT in men (Table 2). Instead, increasing time in deep sleep (sleep stage N3) together with a more severe hypoxic load and older age led to longer RTs being the only parameters included in our regression models. In men, time- and frequency-domain HRV models were identical. In short-term models, however, lower time-domain HRV and lower HF_NU_ together with more severe hypoxic load and longer time spent in N3 were associated with longer RTs in men (Table 2). In addition, the presence of COPD and T2DM was positively associated, while the presence of hypertension was negatively associated with longer RTs. All parameters included in the stepwise regression models are presented in Table 2.

**Table 2.** Stepwise linear regression models for changes in median reaction time due to demographic factors, HRV, sleep, and comorbidities

	Men		Women	
	Overnight	Short term	Overnight	Short term
	β (*SE*)	β (*SE*)	β (*SE*)	β (*SE*)
Time domain				
Age [y]	1.892 (0.649)^*^	1.323 (0.077)^**^	1.148 (0.775)	1.710 (0.093)^**^
BMI [kg/m^2^]	—	0.653 (0.124)^**^	—	0.350 (0.124)
DesSev [%]	10.500 (4.517)^*^	4.461 (0.465)^**^	—	—
*N* of resp. events	—	−1.241 (0.445)	—	−1.823 (0.590)
Mean NN [ms]	—	−21.514 (0.622)^**^	—	−102.226 (9.189)^**^
SDNN [ms]	—	154.680 (35.229)^**^	—	132.865 (54.828)
RMSSD [ms]	—	−234.602 (36.248)^**^	−467.927 (255.124)	−202.035 (44.393)^**^
N1 [min]	—	—	—	−2.844 (1.805)
N2 [min]	—	—	—	—
N3 [min]	0.748 (0.239)^*^	4.961 (0.684)^**^	—	1.251 (0.734)
REM [min]	—	−1.065 (0.619)	—	—
COPD	—	62.516 (3.604)^**^	—	—
T2DM	—	17.136 (2.718)^**^	66.138 (31.379)^*^	53.527 (3.814)^**^
Hypertension	—	−30.194 (2.206)^**^	—	—
Adj. *R*^2^	0.0425	0.0436	0.0307	0.0396
Frequency domain				
Age [y]	1.892 (0.649)^*^	1.255 (0.075)^**^	1.180 (0.778)	1.526 (0.089)^**^
BMI [kg/m^2^]	—	0.792 (0.124)^**^	—	0.626 (0.124)^**^
DesSev [%]	10.500 (4.517)^*^	4.904 (0.464)^**^	—	—
*N* of resp. events	—	−1.362 (0.444)	—	−1.995 (0.591)^**^
HF_NU_	—	−31.767 (4.408)^**^	—	−78.781 (6.806)^**^
LF/HF	—	—	—	−2.346 (0.393)^**^
HFc	—	10.410 (4.160)	—	38.358 (7.152)^**^
N1 [min]	—	—	—	−3.635 (1.802)
N2 [min]	—	—	—	—
N3 [min]	0.748 (0.239)^*^	5.167 (0.679)^**^	—	1.910 (0.733)
REM [min]	—	−0.981 (0.618)	—	—
COPD	—	66.520 (3.554)^**^	—	—
T2DM	—	19.589 (2.669)^**^	67.680 (31.513)^*^	62.873 (3.723)^**^
Hypertension	—	−30.434 (2.193)^**^	—	—
Adj. *R*^2^	0.0425	0.0440	0.0217	0.0390

Abbreviations: Adj. *R*^2^, adjusted *R*^2^ (model fit); BMI, body mass index; COPD, chronic obstructive pulmonary disease; DesSev, desaturation severity; HFc, cross spectral coherence in the HF band; HF_NU_, normalized power in the HF band (HF/(HF + LF)); HRV, heart rate variability; LF/HF, ratio of LF to HF band power; *N* of resp. events, the number of respiratory events within the period of analysis; REM, rapid eye movement sleep; RMSSD, root mean square of successive differences; SDNN, standard deviation of NN intervals; *SE*, standard error of β; T2DM, type II diabetes mellitus; β, estimated coefficient for the regression model.

^*^The statistically significant β-coefficient (*p* < 0.05).

^**^The statistically significant β-coefficient (*p* < 0.001).

In women, age, RMSSD, and T2DM were included in overnight models, but only the presence of T2DM was significantly associated with an RT increase (Table 2). In the short-term models, a lower time-domain HRV, HF_NU_, and LF/HF, and a higher HFc were associated with increased median RTs in the PVT (Table 2). In addition, older age and an increase in the presence of T2DM led to longer RTs. Segment-wise sleep structure and hypoxic load were not associated with RT performance in women.

In men’s overnight models, the same parameters were significantly associated with an increase in the number of lapses in PVT (Supplementary Table S1) as in models for the median RT in PVTs (Table 2). Furthermore, the short-term models for the number of lapses were similar to the median RT-based models, in addition to a reduced time in light sleep (sleep stage N1) being associated with a lower number of lapses. In women, the number of lapses was not significantly linked to HRV, sleep structure, or comorbidities in the overnight models (Supplementary Table S1). In the short-term models, lower time-domain HRV, a reduced HF_NU_, a longer time in N3, and the presence of hypertension together with older age and an increased DesSev led to an increased number of lapses in the PVT.

In men’s overnight regression models for subjective sleepiness (the ESS score), increasing DesSev was the only parameter leading to a significantly higher ESS score (Supplementary Table 2). In the short-term models, the DesSev, time-domain HRV, light sleep structure (N1 and N2), and comorbidities were all associated with the ESS score. As in men, HRV, sleep structure, or comorbidities were not associated with the ESS score in the women’s overnight model (Supplementary Table S2). In the short-term models, anthropometric factors and HRV were associated with the ESS score, but sleep structure and comorbidities were not.

### Patient-wise comparison

**Figure 3.**
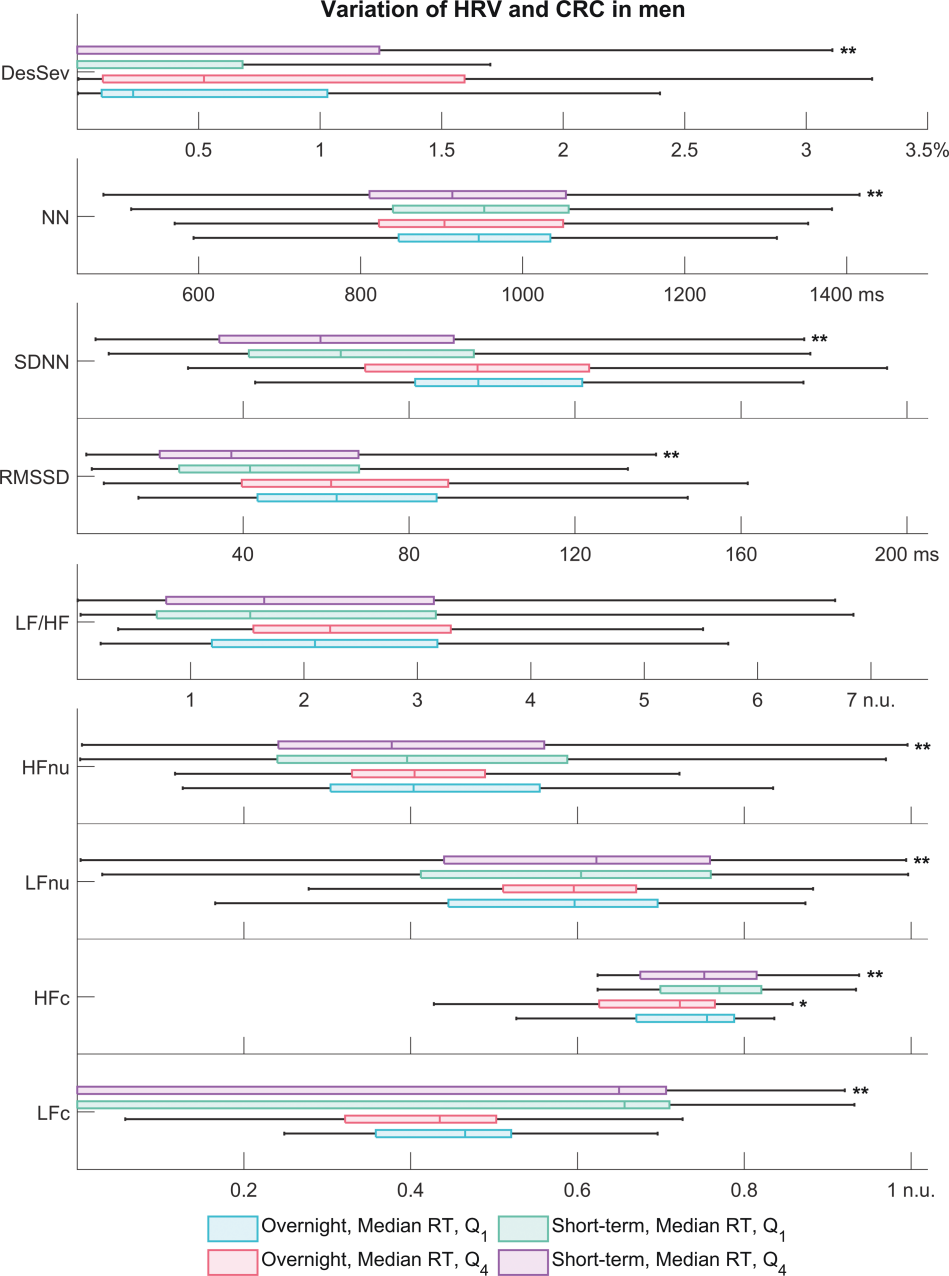
Variation of overnight and short-term heart rate variability (HRV) and cardiorespiratory coupling (CRC) parameters in men based on their performance in psychomotor vigilance task (PVT). In general, the variation within parameters is higher in the short-term data: by averaging the parameter values overnight, we lose important physiological information. Abbreviations: DesSev, desaturation severity; HFc, cross-spectral coherence in the HF band; HFnu, normalized power in the HF band (HF/(LF+HF)); LFc, cross-spectral coherence in the low-frequency band; LF/HF, ratio of LF to HF band power; LFnu, normalized power in the low-frequency band (LF/(LF+HF)); NN, interval between two successive normal R peaks; RMSSD, root mean square of successive differences; SDNN, standard deviation of NN intervals. *Statistically significant difference compared with the vigilant group *Q*_1_ (*p* < 0.05), **statistically significant difference compared with vigilant group *Q*_1_ (*p* < 0.001).

**Figure 4.**
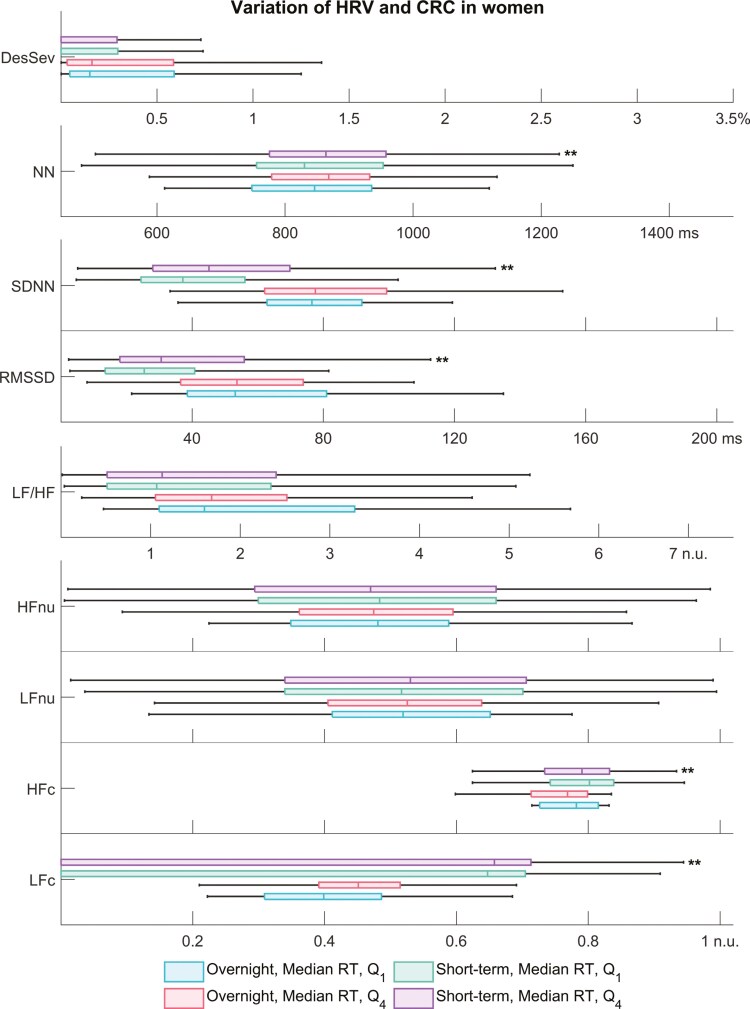
Variation of overnight and short-term heart rate variability (HRV) and cardiorespiratory coupling (CRC) parameters in women based on their performance in psychomotor vigilance task (PVT). In general, the variation within parameters is higher in the short-term data: by averaging the parameter values overnight, we lose important physiological information. Abbreviations: DesSev, desaturation severity; HFc, cross-spectral coherence in the HF band; HFnu, normalized power in the HF band (HF/(LF+HF)); LFc, cross-spectral coherence in the low-frequency band; LF/HF, ratio of LF to HF band power; LFnu, normalized power in the low-frequency band (LF/(LF+HF)); NN, interval between two successive normal R peaks; RMSSD, root mean square of successive differences; SDNN, standard deviation of NN intervals. **Statistically significant difference compared with the vigilant group *Q*_1_ (*p* < 0.001).

When patients were split into quartiles based on the median RT, worse performance in PVT was not significantly linked to OSA severity, overnight HRV, or CRC (Supplementary Table S3). However, we observed an insignificant worsening trend in the OSA severity (i.e. AHI, ODI, T90, and DesSev) toward non-vigilant men (*Q*_4_). In non-vigilant men, only the overnight HFc differed significantly from the well-performing, vigilant group (*Q*_1_, Figure 3). In similar short-term analyses, hypoxic load severity and all HRV parameters except the LF/HF ratio significantly differed between vigilant and non-vigilant men. The findings in women were similar excluding the frequency-domain HRV parameters (Figure 4).

Increasing hypoxic load was linked only with the conventional OSA parameters when dividing patients into four quartiles based on DesSev (Supplementary Table S4). In addition, we observed the lowest HF coherence with the highest variance (*p* < 0.008) among the most hypoxic patients (DesSev *Q*_4_). Finally, when patients were divided into quartiles based on HF_NU_, Increasing HF_NU_ was significantly associated only with other HRV parameters (Supplementary Table S5).

## Discussion

OSA-related hypoxic load contributes to decreased HRV and inferior PVT performance but the association between HRV and PVT performance has remained unrecognized. In this study, we investigated whether HRV and PVT performance are connected among suspected OSA patients and hypothesized that nocturnal short-term HRV is more strongly linked to impaired daytime vigilance compared with overnight HRV. In overnight analyses, HRV was not related to the PVT performance in men or women, but older, more severely hypoxic men with longer N3 durations showed a stronger association with poor PVT performance. However, our new approach to utilize the short-term analysis showed that lower vagal modulation and interbeat variability were linked to worse PVT performance together with age and hypoxic load in men with comorbidities. In women, acutely decreased HRV and T2DM were associated with poor PVT performance. As overnight HRV was not associated with impaired vigilance in our population, but the acutely worsening HRV in terms of decreasing vagal modulation indicated poor PVT performance, the current results importantly suggest that overnight HRV measurements might not be optimal to describe the physiological stress caused by OSA.

The results demonstrate that overnight HRV is not an indicator of poor PVT performance. We obtained the same results when analyzing the relationship between overnight HRV and subjective sleepiness quantified by using the ESS. These findings were against our hypothesis, and they are counterintuitive as sleep deprivation is a common daytime symptom of OSA [4]. Moreover, previous studies [23, 24, 35] have shown that sleep deprivation deteriorates HRV. However, it is noteworthy that none of these studies performed overnight HRV measurements nor included patients with sleep disorders but only healthy participants. In addition, the connection between deteriorated HRV and impaired vigilance was shown with a custom frequency band [23, 35] instead of the conventional frequency bands that we used in our HRV analyses. Although overnight HRV was not associated with impaired vigilance in our study population, the acutely worsening HRV indicated poor PVT performance supporting our hypothesis. In men and women, shorter NN intervals (i.e. higher HR), increased overall variability (SDNN), decreased interbeat variability (RMSSD), and weaker vagal modulation indicated worsening PVT performance in segment-level analyses (Table 2, Supplementary Table S1). The acute (short-term) HRV parameter values also significantly differed between vigilant and non-vigilant patients (Figures 3 and 4).

According to our results, the overnight HRV evaluation might not be optimal for describing the physiological stress caused by OSA. With stable breathing, HRV is a reliable measure of ANS state [17]. During the daytime, OSA patients have increased SNS activity compared with non-OSA controls [36, 37]. In OSA, however, nocturnal breathing becomes unstable, marking poor sleep quality and impaired CRC [20]. Thus, the respiratory events, desaturations, and the resulting cyclical hypo- and hyperventilation (loop gain) may interfere with overnight HRV analyses making the interpretation demanding, as demonstrated in the review by Sequeira et al. [36] presenting inconsistent overnight HRV results in OSA patients. By averaging HRV over long periods of stable and unstable breathing, we lose a significant amount of information on the nocturnal unstable breathing periods and continuous high-magnitude variance. For example, we have previously shown that an increasing hypoxic load leads to short-term sympathetic overactivity and impaired CRC [18, 19], but we did not observe the same effect in our current overnight analyses (Supplementary Table S4). Despite decreased long-term HRV being a good indicator of sympathetic overdrive and, thus, impaired health [17], OSA patients could benefit from complementary, more detailed nocturnal HRV measurements considering the acute, short-term changes in ANS activity and their severity. In addition, overnight and acute HRV measurements could be complemented with other measurements, such as baroreflex sensitivity as it is connected to impaired vagal modulation and objective daytime sleepiness among other effects of OSA [38, 39]. By measuring acute HRV changes and other indices evaluating autonomic modulation, we could obtain a more accurate picture of the nocturnal physiological consequences of respiratory events. Thus, these important aspects warrant further investigation. Nevertheless, with these HRV measurements, we could easily obtain a more comprehensive overview of the physiological effects of OSA as ECG is always recorded as a part of the routine PSG.

In this study, increasing nocturnal hypoxic load, age, and N3 duration were associated with impaired vigilance during overnight analyses in men (Table 2 and Supplementary Table S1). We observed an increasing but insignificant trend in hypoxic load severity and conventional OSA severity parameters when men were grouped based on the median RT (Figure 3, Supplementary Table S3). In addition, the hypoxic load severity was the only significant factor indicating subjective sleepiness in men (Supplementary Table S2). The number of respiratory events, however, was not linked to poor PVT performance in the regression analyses (Table 2 and Supplementary Table S1). Numerous earlier studies [3, 11–16, 40] support our findings demonstrating that increasing hypoxic load severity is more strongly connected to impaired vigilance and objective daytime sleepiness in OSA patients than the AHI, especially in men. Furthermore, a more severe hypoxic load has been more strongly linked, for example, to sympathetic overactivity, an increased risk for cardiovascular diseases, and other comorbidities [40, 41]. As the hypoxic load is one of the key factors contributing to a decreased ability to sustain attention and to other physiological consequences, OSA patients could benefit from a more detailed analysis of the oxygen saturation profile, considering intermittent desaturations and tonic hypoxic conditions.

Significantly, the N3 duration increased with a more severe hypoxic load leading to worse PVT performance (Table 2 and Supplementary Table S1). N3 sleep is essential for memory consolidation and the glymphatic clearance of metabolic waste products from the brain [42–45]. Furthermore, the respiratory event rate is reduced in N3 compared with other sleep stages due to higher upper airway muscle activity and arousal threshold [46, 47]. In OSA, N3 sleep is usually the period with no respiratory events and hypoxic load, and its duration decreases as OSA worsens. Thus, our current results seem to demonstrate that the ability to sustain attention and body recovery functions suffers more with a more severe hypoxic load and increased duration of N3 sleep. However, we did not concentrate on this specific topic in our analyses, and it needs to be explored in more detail. In addition, our study demonstrated the typical differences in the effects of hypoxic load on vigilance and subjective sleepiness between men and women: in women, we did not observe a similar connection between hypoxic load and PVT performance as seen in men. Men are more prone to experiencing more respiratory events as well as longer and deeper desaturations with longer recovery times for anatomical and physiological reasons [48, 49], which partly explains the sex differences. In addition, women tend to have a less sensitive peripheral chemoreflex to the hypoxic stimulus [50], and their PVT performance seems to be affected by arousals rather than nocturnal hypoxic load [14].

### Strengths and limitations

This study has several strengths. First, it benefitted from a substantial sample size, which enhanced the statistical power of this study and allowed for more robust analyses. Additionally, the inclusion of both sexes in this research provided a more comprehensive and representative dataset, revealing sex-specific patterns in the results. The dataset also included approximately 50:50 men and women, which is unusual compared with the majority of OSA studies. Moreover, this study’s statistical methods were well-conceived and rigorously applied. This research adopted an interdisciplinary approach incorporating elements from diverse fields such as sleep medicine, cardiology, and psychomotor vigilance.

Despite its strengths, this study also has certain limitations. For instance, the PVTs were performed on the evening preceding the PSG. This setup complicates the interpretation of the relationship between the level of vigilance and sleep as the pre-PVT circumstances have not been controlled: routine daily activities could affect the PVT performance in addition to the quality of the previous night’s sleep. However, Djonlagic et al. [51] showed that patients with untreated OSA present no differences between the PVTs conducted in the evening before and the morning after the PSG. Therefore, we believe that this setup better captures the overall vigilance level in the patient’s normal circumstances at home. Ideally, the PVT could, for example, also be conducted after the PSG or repeatedly in a couple of mornings to reduce a common first-night effect in the PSG potentially affecting the sleep quality and thus the other measurements such as the PVT. On a large scale, this might be possible to implement using home-PSG or simplified wearable sleep monitoring systems in combination with mobile PVT applications.

In addition, our dataset did not include sleep diaries and had limited information related to patients’ caffeine intake and alcohol consumption. This lack of information is a limitation because chronic sleep deprivation, caffeine intake, and alcohol consumption affect neurocognitive performance and vagal modulation [35, 39, 52]. Patients were merely instructed to treat the day preceding the PSG as a regular day; this way, the measurement setup provides a more realistic overview of the patients’ sleep quality and daily vigilance. Furthermore, we had incomplete medication data for the studied population. Certain medications, such as psychoactive drugs and β-blockers, can affect vigilance, vagal modulation, and thus cardiac functioning. However, we excluded patients with arrhythmias and a history of cardiac failure and adjusted the stepwise linear regression models with the available and relevant comorbidities.

### Clinical implications

Short-term HRV shows potential for establishing a more robust correlation with daytime vigilance, enabling the detection of subtle variations in autonomic regulation. Additionally, by utilizing short-term HRV analysis, healthcare providers may identify early signs of sleep-related health risks, such as cardiovascular issues or disruptions in autonomic function. This approach has the potential to enhance patient care and contribute to early intervention, ultimately improving health outcomes. While these benefits hold promise for both research and clinical applications, further research is necessary to explore its full implications. Nevertheless, this study provides important information on how the data from PSG recordings could be analyzed more comprehensively in the future to improve personalized treatment paths and reduce the risks due to untreated OSA.

## Conclusions

The hypoxic load and its severity are key indicators of impaired vigilance in men while the overnight HRV changes were not associated with PVT performance. However, by studying physiological changes in our novel short-term methodological manner, we observed that acutely impaired HRV relates to poor PVT performance in both men and women. These novel findings emphasize that we lose a marked amount of information on vagal modulation and other physiological consequences in OSA patients due to unstable breathing patterns by averaging HRV overnight. As acutely impaired HRV relates to impaired vigilance and markers indicating increased cardiac load and sympathetic activity, it would be important to study the nocturnal HRV changes as a time series instead of an overnight average. This novel approach could provide a more detailed picture of the state of the cardiac and nervous systems in OSA patients.

## Supplementary Material

Supplementary material is available at *SLEEP* online.

zsae282_suppl_Supplementary_Tables_S1-S5

## Data Availability

The data include medical records and personal information and therefore the data can only be shared within the confinements of the Australian legislation and ethical conventions. Reasonable requests considering data sharing will be individually assessed.

## References

[1] Benjafield AV, Ayas NT, Eastwood PR, et al Estimation of the global prevalence and burden of obstructive sleep apnoea: a literature-based analysis. Lancet Respir Med. 2019;7:687–698. doi: https://doi.org/10.1016/S2213-2600(19)30198-5 31300334 PMC7007763

[2] Lyons MM, Bhatt NY, Pack AI, Magalang UJ. Global burden of sleep-disordered breathing and its implications. Respirology. 2020;25:690–702. doi: https://doi.org/10.1111/resp.13838 32436658

[3] Sforza E, Haba-Rubio J, De Bilbao F, Rochat T, Ibanez V. Performance vigilance task and sleepiness in patients with sleep-disordered breathing. Eur Respir J. 2004;24:279–285. doi: https://doi.org/10.1183/09031936.04.00091903 15332398

[4] Batool-Anwar S, Kales SN, Patel SR, Varvarigou V, DeYoung PN, Malhotra A. Obstructive sleep apnea and psychomotor vigilance task performance. Nat Sci Sleep. 2014;6:65–71. doi: https://doi.org/10.2147/NSS.S53721 24920941 PMC4043718

[5] Hillman D, Mitchell S, Streatfeild J, Burns C, Bruck D, Pezzullo L. The economic cost of inadequate sleep. Sleep. 2018;41. doi: https://doi.org/10.1093/sleep/zsy083 29868785

[6] Dewan NA, Nieto FJ, Somers VK. Intermittent hypoxemia and OSA: implications for comorbidities. Chest. 2015;147:266–274. doi: https://doi.org/10.1378/chest.14-0500 25560865 PMC4285080

[7] Arnardottir ES, Bjornsdottir E, Olafsdottir KA, Benediktsdottir B, Gislason T. Obstructive sleep apnoea in the general population: highly prevalent but minimal symptoms. Eur Respir J. 2016;47:194–202. doi: https://doi.org/10.1183/13993003.01148-2015 26541533

[8] Dinges DF, Powell JW. Microcomputer analyses of performance on a portable, simple visual RT task during sustained operations. Behav Res Methods Instrum Comput. 1985;17:652–655. doi: https://doi.org/10.3758/bf03200977

[9] Basner M, Hermosillo EBA, Nasrini JBS, et al Repeated administration effects on psychomotor vigilance test performance. Sleep. 2018;41. doi: https://doi.org/10.1093/sleep/zsx187 29126328

[10] Lee IS, Bardwell WA, Ancoli-Israel S, Dimsdale JE. Number of lapses during the psychomotor vigilance task as an objective measure of fatigue. J Clin Sleep Med. 2010;6:163–168. doi: https://doi.org/10.5664/jcsm.27766 20411694 PMC2854704

[11] Tanno S, Tanigawa T, Maruyama K, Eguchi E, Abe T, Saito I. Sleep-related intermittent hypoxia is associated with decreased psychomotor vigilance in Japanese community residents. Sleep Med. 2017;29:7–12. doi: https://doi.org/10.1016/j.sleep.2016.08.024 28153218

[12] Kainulainen S, Töyräs J, Oksenberg A, et al Severity of desaturations reflects OSA-related daytime sleepiness better than AHI. J Clin Sleep Med. 2019;15:1135–1142. doi: https://doi.org/10.5664/jcsm.7806 31482835 PMC6707054

[13] Kainulainen S, Duce B, Korkalainen H, et al Severe desaturations increase psychomotor vigilance task-based median reaction time and number of lapses in obstructive sleep apnoea patients. Eur Respir J. 2020;55:1901849. doi: https://doi.org/10.1183/13993003.01849-2019 32029446 PMC7142879

[14] Pahari P, Korkalainen H, Karhu T, et al Reaction time in psychomotor vigilance task is related to hypoxic load in males with sleep apnea. J Sleep Res. 2024;33:e13988. doi: https://doi.org/10.1111/jsr.13988 37448111

[15] Pahari P, Korkalainen H, Karhu T, et al Obstructive sleep apnea-related intermittent hypoxaemia is associated with impaired vigilance. J Sleep Res. 2023;32:e13803. doi: https://doi.org/10.1111/jsr.13803 36482788

[16] Karhu T, Leppänen T, Korkalainen H, et al Desaturation event scoring criteria affect the perceived severity of nocturnal hypoxic load. Sleep Med. 2022;100:479–486. doi: https://doi.org/10.1016/j.sleep.2022.09.024 36257201

[17] Shaffer F, Ginsberg JP. An overview of heart rate variability metrics and norms. Front Public Health. 2017;5:258. doi: https://doi.org/10.3389/fpubh.2017.00258 29034226 PMC5624990

[18] Hietakoste S, Karhu T, Sillanmäki S, et al OSA-related respiratory events and desaturation severity are associated with the cardiac response. ERJ Open Res. 2022;8:00121-2022. doi: https://doi.org/10.1183/23120541.00121-2022 36299363 PMC9589326

[19] Hietakoste S, Armanac-Julian P, Karhu T, et al Acute cardiorespiratory coupling impairment in worsening sleep apnea-related intermittent hypoxemia. IEEE Trans Biomed Eng. 2024;71:326–333. doi: https://doi.org/10.1109/TBME.2023.3300079 37523277

[20] Thomas RJ, Wood C, Bianchi MT. Cardiopulmonary coupling spectrogram as an ambulatory clinical biomarker of sleep stability and quality in health, sleep apnea, and insomnia. Sleep. 2018;41. doi: https://doi.org/10.1093/sleep/zsx196 PMC6018901 29237080

[21] Thomas RJ, Mietus JE, Peng CK, Goldberger AL. An electrocardiogram-based technique to assess cardiopulmonary coupling during sleep. Sleep. 2005;28:1151–1161. doi: https://doi.org/10.1093/sleep/28.9.1151 16268385

[22] Varon C, Caicedo A, Testelmans D, Buyse B, Van Huffel S. A novel algorithm for the automatic detection of sleep apnea from single-lead ECG. IEEE Trans Biomed Eng. 2015;62:2269–2278. doi: https://doi.org/10.1109/TBME.2015.2422378 25879836

[23] Chua ECP, Tan WQ, Yeo SC, et al Heart rate variability can be used to estimate sleepiness-related decrements in psychomotor vigilance during total sleep deprivation. Sleep. 2012;35:325–334. doi: https://doi.org/10.5665/sleep.1688 22379238 PMC3274333

[24] Bourdillon N, Jeanneret F, Nilchian M, Albertoni P, Ha P, Millet GP. Sleep deprivation deteriorates heart rate variability and photoplethysmography. Front Neurosci. 2021;15:642548. doi: https://doi.org/10.3389/fnins.2021.642548 33897355 PMC8060636

[25] Qin H, Steenbergen N, Glos M, et al The different facets of heart rate variability in obstructive sleep apnea. Front Psychiatry. 2021;12:1–20. doi: https://doi.org/10.3389/fpsyt.2021.642333 PMC8339263 34366907

[26] Berry RB, Brooks R, Gamaldo CE, et al The AASM manual for the scoring of sleep and associated events: rules, terminology and technical specifications. Am Acad Sleep Med. 2017(Version 2.4.):1–89. doi: https://doi.org/10.1016/j.carbon.2012.07.027

[27] AASM. Sleep-related breathing disorders in adults: recommendations for syndrome definition and measurement techniques in clinical research. Sleep. 1999;22:667–689. doi: https://doi.org/10.1093/sleep/22.5.667 10450601

[28] Mueller ST, Piper BJ. The Psychology Experiment Building Language (PEBL) and PEBL Test Battery. J Neurosci Methods. 2014;222:250–259. doi: https://doi.org/10.1016/j.jneumeth.2013.10.024 24269254 PMC3897935

[29] Tarvainen MP, Niskanen JP, Lipponen JA, Ranta-aho PO, Karjalainen PA. Kubios HRV—heart rate variability analysis software. Comput Methods Programs Biomed. 2014;113:210–220. doi: https://doi.org/10.1016/j.cmpb.2013.07.024 24054542

[30] Pan J, Tompkins WJ. A real-time QRS detection algorithm. IEEE Trans Biomed Eng. 1985;32:230–236. doi: https://doi.org/10.1109/TBME.1985.325532 3997178

[31] Kulkas A, Tiihonen P, Julkunen P, Mervaala E, Töyräs J. Novel parameters indicate significant differences in severity of obstructive sleep apnea with patients having similar apnea-hypopnea index. Med Biol Eng Comput. 2013;51:697–708. doi: https://doi.org/10.1007/s11517-013-1039-4 23417543

[32] Tarvainen MP, Ranta-aho PO, Karjalainen PA. An advanced detrending method with application to HRV analysis. IEEE Trans Biomed Eng. 2002;49:172–175. doi: https://doi.org/10.1109/10.979357 12066885

[33] Malik M, Bigger JT, Camm AJ, et al Heart rate variability. Standards of measurement, physiological interpretation, and clinical use. Task Force of the European Society of Cardiology and the North American Society of Pacing and Electrophysiology. Circulation. 1996;93:1043–1065. doi: https://doi.org/10.1161/01.CIR.93.5.1043 8598068

[34] Orini M, Bailon R, Mainardi LT, Laguna P, Flandrin P. Characterization of dynamic interactions between cardiovascular signals by time-frequency coherence. IEEE Trans Biomed Eng. 2012;59:663–673. doi: https://doi.org/10.1109/TBME.2011.2171959 22155936

[35] Henelius A, Sallinen M, Huotilainen M, Müller K, Virkkala J, Puolamäki K. Heart rate variability for evaluating vigilant attention in partial chronic sleep restriction. Sleep. 2014;37:1257–1267. doi: https://doi.org/10.5665/sleep.3850 24987165 PMC4074968

[36] Sequeira VCC, Bandeira PM, Azevedo JCM. Heart rate variability in adults with obstructive sleep apnea: a systematic review. Sleep Sci. 2019;12:214–221. doi: https://doi.org/10.5935/1984-0063.20190082 31890098 PMC6932836

[37] Urbanik D, Gać P, Martynowicz H, et al Obstructive sleep apnea as a predictor of reduced heart rate variability. Sleep Med. 2019;54:8–15. doi: https://doi.org/10.1016/j.sleep.2018.09.014 30529071

[38] Lombardi C, Parati G, Cortelli P, et al Daytime sleepiness and neural cardiac modulation in sleep-related breathing disorders. J Sleep Res. 2008;17:263–270. doi: https://doi.org/10.1111/j.1365-2869.2008.00659.x 18503513

[39] Jordan AS, Mcsharry DG, Malhotra A. Adult obstructive sleep apnoea. Lancet. 2014;383:736–747. doi: https://doi.org/10.1016/S0140-6736(13)60734-5 23910433 PMC3909558

[40] Korkalainen H, Kainulainen S, Sigridur Islind A, et al Review and perspective on sleep-disordered breathing research and translation to clinics. Sleep Med Rev. 2024;73:101874. doi: https://doi.org/10.1016/j.smrv.2023.101874 38091850

[41] Floras JS. Sleep apnea and cardiovascular disease: an enigmatic risk factor. Circ Res. 2018;122:1741–1764. doi: https://doi.org/10.1161/CIRCRESAHA.118.310783 29880501

[42] Marshall L, Born J. The contribution of sleep to hippocampus-dependent memory consolidation. Trends Cogn Sci. 2007;11:442–450. doi: https://doi.org/10.1016/j.tics.2007.09.001 17905642

[43] Diekelmann S, Born J. The memory function of sleep. Nat Rev Neurosci. 2010;11:114–126. doi: https://doi.org/10.1038/nrn2762 20046194

[44] Fultz NE, Bonmassar G, Setsompop K, et al Coupled electrophysiological, hemodynamic, and cerebrospinal fluid oscillations in human sleep. Science. 2019;366:628–631. doi: https://doi.org/10.1126/science.aax5440 31672896 PMC7309589

[45] Reddy OC, van der Werf YD. The sleeping brain: harnessing the power of the glymphatic system through lifestyle choices. Brain Sci. 2020;10:868. doi: https://doi.org/10.3390/brainsci10110868 33212927 PMC7698404

[46] Ratnavadivel R, Stadler D, Windler S, et al Upper airway function and arousability to ventilatory challenge in slow wave versus stage 2 sleep in obstructive sleep apnoea. Thorax. 2010;65:107–112. doi: https://doi.org/10.1136/thx.2008.112953 19850964

[47] Kim SH, Yang CJ, Baek JT, et al Does rapid eye movement sleep aggravate obstructive sleep apnea? Clin Exp Otorhinolaryngol. 2019;12:190–195. doi: https://doi.org/10.21053/ceo.2018.00934 30415523 PMC6453783

[48] Leppänen T, Kulkas A, Duce B, Mervaala E, Töyräs J. Severity of individual obstruction events is gender dependent in sleep apnea. Sleep Breath. 2017;21:397–404. doi: https://doi.org/10.1007/s11325-016-1430-0 27966055

[49] Rowley JA, Sanders CS, Zahn BR, Badr MS. Gender differences in upper airway compliance during NREM sleep: role of neck circumference. J Appl Physiol (1985). 2002;92:2535–2541. doi: https://doi.org/10.1152/japplphysiol.00553.2001 12015370

[50] Caravita S, Faini A, Lombardi C, et al Sex and acetazolamide effects on chemoreflex and periodic breathing during sleep at altitude. Chest. 2015;147:120–131. doi: https://doi.org/10.1378/chest.14-0317 25188815

[51] Djonlagic I, Guo M, Matteis P, Carusona A, Stickgold R, Malhotra A. First night of CPAP: impact on memory consolidation attention and subjective experience. Sleep Med. 2015;16:697–702. doi: https://doi.org/10.1016/j.sleep.2015.01.017 25953301 PMC5238960

[52] Wong KKH, Marshall NS, Grunstein RR, Dodd MJ, Rogers NL. Comparing the neurocognitive effects of 40 h sustained wakefulness in patients with untreated OSA and healthy controls. J Sleep Res. 2008;17:322–330. doi: https://doi.org/10.1111/j.1365-2869.2008.00665.x 18522688

