# Supplementary material for: Nocturnal short-term heart rate variability reflects impaired daytime vigilance better than overnight heart rate variability in suspected obstructive sleep apnea patients
Source: Sleep. 2024 Dec 13;48(4):zsae282. doi: 10.1093/sleep/zsae282 (PMC11985393; doi:10.1093/sleep/zsae282)
Supplement: zsae282_suppl_Supplementary_Tables_S1-S5 [file zsae282_suppl_supplementary_tables_s1-s5.pdf]

Nocturnal short-term HRV reflects impaired daytime vigilance better than overnight HRV in suspected OSA patients

Salla Hietakoste[1,2]*, Tuomas Karhu[1,2], Carolina Lombardi[3,4], Pablo Armañac-Julián[5,6], Raquel Bailón[5,6], Brett Duce[7,8], Saara Sillanmäki[2,9], Juha Töyräs[1,10,11], Timo Leppänen[1,2,11], Sami Myllymaa[1,2], Samu Kainulainen[1,2]

[1]Department of Technical Physics, University of Eastern Finland, Kuopio, Finland
[2]Diagnostic Imaging Center, Kuopio University Hospital, Kuopio, Finland
[3]Sleep Disorders Center and Department of Cardiology, Neural and Metabolic Sciences, San Luca Hospital, IRCCS Istituto Auxologico Italiano, Milan, Italy
[4]School of Medicine and Surgery, University of Milano-Bicocca, Milan, Italy
[5]Centro de Investigación Biomédica en Red en Bioingeniería, Biomateriales y Nanomedicina (CIBER-BBN), Instituto de Salud Carlos III, Madrid, Spain
[6]Biomedical Signal Interpretation and Computational Simulation (BSICoS) Group, Aragón Institute of Engineering Research (I3A), University of Zaragoza, Zaragoza, Spain
[7]Sleep Disorders Centre, Department of Respiratory and Sleep Medicine, Princess Alexandra Hospital, Brisbane, Australia
[8]Institute for Health and Biomedical Innovation, Queensland University of Technology, Brisbane, Australia
[9]Institute of Clinical Medicine, University of Eastern Finland, Kuopio, Finland
[10]Science Service Center, Kuopio University Hospital, Kuopio, Finland
[11]School of Electrical Engineering and Computer Science, The University of Queensland, Brisbane, Australia

Corresponding author:
Salla Hietakoste
Department of Technical Physics, University of Eastern Finland,
Yliopistonrinne 3, P.O. BOX 1627 (Canthia), FI-70211 Kuopio, Finland
salla.hietakoste@uef.fi

Table S1. Stepwise linear regression models for changes in the number of lapses due to demographic factors, HRV, sleep, and comorbidities.

	Men		Women	
	**Overnight**	**Short-term**	**Overnight**	**Short-term**
	**β (SE)**	**β (SE)**	**β (SE)**	**β (SE)**
**Time-domain**				
**Age [y]**	0.283 (0.118)*	0.200 (0.014)**	0.285 (0.140)*	0.225 (0.017)**
**BMI [kg*m^{-2}]**	-	0.072 (0.022)	-	-0.080 (0.022)**
**DesSev [%]**	1.985 (0.843)*	0.840 (0.084)**	-	0.881 (0.174)**
**N of resp. events**	-	-0.219 (0.087)	-	-
**Mean NN [ms]**	-	-3.043 (1.116)	-22.479 (14.801)	-19.104 (1.619)**
**SDNN [ms]**	-	35.774 (6.333)**	-	38.323 (9.771)**
**RMSSD [ms]**	-	-47.136 (6.411)**	-	-36.578 (7.781)**
**N1 [min]**	-0.091 (0.051)	-1.361 (0.213)**	-	-0.733 (0.302)
**N2 [min]**	-	-	-	0.330 (0.125)
**N3 [min]**	0.103 (0.047)*	0.715 (0.126)**	0.064 (0.043)	0.666 (0.142)**
**REM [min]**	-0.088 (0.056)	-0.353 (0.116)	-	-
**COPD**	-	4.553 (0.646)**	-	2.601 (0.847)
**T2DM**	-	6.494 (0.487)**	-	2.078 (0.666)
**Hypertension**	-	-4.818 (0.396)**	-	2.782 (0.480)**
**Adj. R^2**	**0.0433**	**0.0332**	**0.0143**	**0.0245**
**Frequency-domain**				
**Age [y]**	0.283 (0.118)*	0.184 (0.013)**	0.211 (0.135)	0.178 (0.016)**
**BMI [kg*m^{-2}]**	-	0.082 (0.022)**	-	-0.034 (0.022)
**DesSev [%]**	1.985 (0.843)*	0.887 (0.082)**	3.203 (2.237)	0.956 (0.174)**
**N of resp. events**	-	-0.227 (0.067)	-	-
**HF_{NU}**	-	-3.949 (0.793)**	-	-9.644 (1.165)**
**LF/HF**	-	-	-	-0.224 (0.067)**
**HFc**	-	-	-	4.631 (1.233)**
**N1 [min]**	-0.091 (0.051)	-1.341 (0.213)**	-	-0.907 (0.299)
**N2 [min]**	-	-	-	-
**N3 [min]**	0.103 (0.047)*	0.697 (0.125)**	0.069 (0.043)	0.550 (0.125)**
**REM [min]**	-0.088 (0.056)	-0.336 (0.116)	-	-
**COPD**	-	5.016 (0.639)**	-	3.897 (0.837)**
**T2DM**	-	6.785 (0.480)**	-	3.736 (0.648)**
**Hypertension**	-	-4.887 (0.394)**	-	2.470 (0.480)**
**Adj. R^2**	**0.0433**	**0.0319**	**0.0134**	**0.0203**

Abbreviations: HRV = Heart rate variability, β = Estimated coefficient for the regression model, SE = Standard error of β, Adj. R^2 = Adjusted R^2 (model fit), BMI = Body mass index, DesSev = Desaturation severity, N of resp. events = The number of respiratory events within the period of analysis, SDNN = Standard deviation of NN intervals, RMSSD = Root mean square of successive differences, REM = Rapid eye movement sleep, HF_{NU} = Normalized power in the high-frequency band (HF/(HF+LF)), LF/HF = Ratio of low-frequency (LF) to high-frequency band power, HFc = Cross spectral coherence in the high-frequency band, COPD = Chronic obstructive pulmonary disease, T2DM = Type II diabetes mellitus.

* = the statistically significant β-coefficient ($p < 0.05$).
** = the statistically significant β-coefficient ($p < 0.001$).

Table S2. Stepwise linear regression models for changes in the Epworth Sleepiness Scale (ESS) score due to demographic factors, HRV, sleep, and comorbidities.

	Men		Women	
	**Overnight β (SE)**	**Short-term β (SE)**	**Overnight β (SE)**	**Short-term β (SE)**
**Time-domain**				
**Age [y]**	-	-	-	-0.023 (0.003)**
**BMI [kg*m^{-2}]**	-	-	0.086 (0.034)*	0.077 (0.004)**
**DesSev [%]**	0.323 (0.163)*	0.184 (0.017)**	-	-0.171 (0.034)**
**N of resp. events**	-	-0.036 (0.018)	-	-
**Mean NN [ms]**	-	0.341 (0.224)	-	-0.709 (0.295)
**SDNN [ms]**	-	13.698 (1.327)**	-	-14.033 (1.760)**
**RMSSD [ms]**	-	-11.509 (1.343)**	-	14.255 (1.438)**
**N1 [min]**	-	-0.222 (0.043)**	-	0.089 (0.057)
**N2 [min]**	0.013 (0.007)	0.111 (0.021)**	-	-
**N3 [min]**	-	0.0750 (0.027)	-	-
**REM [min]**	-	-	-	0.059 (0.025)
**COPD**	-	0.871 (0.129)**	-	-0.263 (0.160)
**T2DM**	-	0.549 (0.098)**	-	-
**Hypertension**	-0.999 (0.674)	-1.074 (0.077)**	-	0.256 (0.089)
**Adj. R^2**	**0.0207**	**0.0237**	**0.0201**	**0.0323**
**Frequency-domain**				
**Age [y]**	-	-0.005 (0.003)	-	-0.026 (0.003)**
**BMI [kg*m^{-2}]**	-	-0.012 (0.005)	0.090 (0.034)*	0.079 (0.004)**
**DesSev [%]**	0.323 (0.163)*	0.174 (0.015)**	-	-0.236 (0.042)**
**N of resp. events**	-	-	-	0.035 (0.024)
**HF_{NU}**	-	-0.409 (0.161)	-	1.372 (0.219)**
**LF/HF**	-	-	-0.336 (0.208)	-0.028 (0.013)
**HFc**	-	-0.441 (0.154)	-	-0.852 (0.234)**
**N1 [min]**	-	-0.246 (0.038)**	-	-
**N2 [min]**	0.013 (0.007)	0.063 (0.019)**	-	-
**N3 [min]**	-	-	-	-
**REM [min]**	-	-	-	0.046 (0.025)
**COPD**	-	0.787 (0.132)**	-	-
**T2DM**	-	0.486 (0.099)**	-	0.226 (0.122)
**Hypertension**	-0.999 (0.674)	-1.135 (0.081)**	-	0.150 (0.090)
**Adj. R^2**	**0.0207**	**0.0198**	**0.0263**	**0.0318**

Abbreviations: HRV = Heart rate variability, β = Estimated coefficient for the regression model, SE = Standard error of β, Adj. R^2 = Adjusted R^2 (model fit), BMI = Body mass index, DesSev = Desaturation severity, N of resp. events = The number of respiratory events within the period of analysis, SDNN = Standard deviation of NN intervals, RMSSD = Root mean square of successive differences, REM = Rapid eye movement sleep, HF_{NU} = Normalized power in the high-frequency band (HF/(HF+LF)), LF/HF = Ratio of low-frequency (LF) to high-frequency band power, HFc = Cross spectral coherence in the high-frequency band, COPD = Chronic obstructive pulmonary disease, T2DM = Type II diabetes mellitus.

* = the statistically significant β-coefficient ($p < 0.05$).
** = the statistically significant β-coefficient ($p < 0.001$).

Table S3. HRV and CRC in groups based on median reaction time in PVT.

		Men				Women			
		**Q_1**	**Q_2**	**Q_3**	**Q_4**	**Q_1**	**Q_2**	**Q_3**	**Q_4**
**N of patients**		73	71	72	72	66	63	66	63
**Age [y]**		44.9	53.3	52.6	56.1*	49.1	48.4	53.1	55.1
**BMI [kg/m²]**		31.6	32.9	33.0	32.5	36.1	36.1	36.9	34.4
**TST [min]**		420.5	429.5	432.0	422.5	416.5	425.0	432.0	425.5
**Sleep Stages**	**N1 [min]**	44.5	42.0	44.8	43.3	26.8	28.0	28.8	22.5
	**N2 [min]**	160.0	168.0	167.5	149.8	165.0	175.0	161.8	158.5
	**N3 [min]**	54.0	39.0	42.5	59.0	82.5	73.5	72.3	81.0
	**REM [min]**	60.5	49.5	54.3	50.3	66.5	67.0	59.8	61.0
**AHI [events/h]**		13.7	16.9	18.9	19.4	8.3	8.6	9.1	9.9
**ODI [events/h]**		9.7	11.6	14.0	16.9	5.9	4.9	7.7	7.1
**T90 [min]**		3.1	5.8	10.3	11.2	1.7	1.1	2.3	1.5
**DesSev [%]**		0.230	0.403	0.426	0.522	0.125	0.136	0.177	0.156
**PVT**	**Median RT [ms]**	-	-	-	-	-	-	-	-
	**Lapses**	**3**	**7**	**12**	**53.5**	**4**	**12**	**27**	**74**
**ESS**		10	9	9	11	9.5	10	10	12
**Time-domain HRV**	**Mean NN [ms]**	945.8	948.2	916.7	903.4	896.0	883.2	863.0	871.1
	**SDNN [ms]**	96.7	100.3	85.4†	96.5	89.3	82.3	83.9	76.5
	**RMSSD [ms]**	62.5	65.9	57.0	61.2	60.6	55.7	57.2	49.0
	**pNN50 [%]**	14.3	13.0	6.0*	8.0	11.0	14.1	5.8	8.2
**Freq.-domain HRV**	**HF [ms²]**	800	1300	600	500	1100	800	700	1700
	**LF [ms²]**	1300	1500	900	1100	1500	1100	700	2600
	**HF_{NU}**	0.404	0.407	0.431	0.405	0.487	0.534	0.477	0.474
	**LF_{NU}**	0.597	0.593	0.569	0.596	0.513	0.466	0.523	0.526
	**LF/HF**	2.098	1.973	1.945	2.231	1.426	1.235	1.749	1.674
	**HF_{NU} var**	0.022	0.024	0.026	0.027	0.026	0.023	0.025	0.028
**CRC**	**HFc**	0.755	0.750	0.738	0.723	0.785	0.787	0.770	0.773
	**LFc**	0.465	0.430	0.435	0.435	0.426	0.426	0.457	0.428
	**HFc var**	0.024	0.016	0.021†	0.034	0.012	0.010	0.015	0.016

HRV and CRC parameter values are calculated overnight from RR intervals and nasal pressure. Data are presented as median for each quartile and the statistical significance of the difference between quartiles was assessed with the Mann-Whitney U-test. Abbreviations: HRV = Heart rate variability, CRC = Cardiorespiratory coupling measured as the spectral coherence between NN intervals and nasal pressure, PVT = Psychomotor vigilance task, BMI = Body mass index, TST = Total sleep time, REM = Rapid eye movement, AHI = Apnea-hypopnea index, ODI = Oxygen desaturation index, T90 = Time spent in oxygen desaturation < 90%, DesSev = Desaturation severity, RT = Reaction time, ESS = Epworth Sleepiness Scale, SDNN = standard deviation of NN intervals (artifacts corrected), RMSSD = Root mean square of successive differences, pNN50 = Proportion of adjacent NN intervals differing more than 50 ms, HF = High-frequency band (0.15-0.4 Hz), LF = Low-frequency band (0.04-0.15 Hz), HF_{NU} = Normalized power in the HF band (HF/(HF+LF)), LF_{NU} = Normalized power in the LF band (LF/(HF+LF)), LF/HF = Ratio of low-frequency to high-frequency band power, var = variance, HFc = Cross spectral coherence in HF band, LFc = Cross spectral coherence in LF band.

* = statistically significant difference compared to Q_1 ($p < 0.008$). † = statistically significant difference compared to Q_2 ($p < 0.008$), ‡ = statistically significant difference compared to Q_3 ($p < 0.008$). Bolded values denote statistically significant differences compared to all other quartiles ($p < 0.008$).

Table S4. HRV, CRC, and performance in PVT in groups based on DesSev.

		Men				Women			
		Q_1	Q_2	Q_3	Q_4	Q_1	Q_2	Q_3	Q_4
**N of patients**		72	72	72	72	65	64	64	65
**Age [y]**		45.2	54.1*	55.2*	51.5	**42.2**	50.0*	52.8*†	56.3*
**BMI [kg/m^2]**		**27.7**	31.7*	34.3*†	36.0*†	**31.0**	35.8*	38.3*	39.1*
**TST [min]**		429.5	432.5	416.5	416.7	427.5	428.5	424.2	416.5
**Sleep Stages**	**N1 [min]**	33.5	40.0	43.3	**71.5**	22.0	27.0	28.3	30.0
	**N2 [min]**	179.8	165.8	159.0	141.8*†	165.5	166.5	169.3	151.5
	**N3 [min]**	63.0	45.3	51.8	33.3*	81.5	82.3	71.0	76.0
	**REM [min]**	70.3	52.8	53.5*	41.5*	68.0	66.0	67.0	**46.5**
**AHI [events/h]**		**5.3**	**12.7**	**23.5**	**46.9**	**2.4**	**6.2**	**12.7**	**28.1**
**ODI [events/h]**		**1.7**	**8.7**	**21.7**	**49.8**	**1.1**	**3.8**	**11.4**	**29.6**
**T90 [min]**		**0.1**	**1.9**	**11.7**	**79.2**	**0.1**	**0.5**	**4.0**	**42.1**
**DesSev [%]**		-	-	-	-	-	-	-	-
**PVT**	**Median RT [ms]**	356.5	352.5	361	370	396	395.5	394	417
	**Lapses**	8	7	11	11	14	15	16	23
**ESS**		10	10.5	10	10	12	9.5	11	10
**Time-domain HRV**	**Mean NN [ms]**	993.7	948.4	902.2*	852.7*†	873.3	894.7	860.3	848.2
	**SDNN [ms]**	101.5	95.3	91.5	93.2	88.4	85.7	75.7	82.6
	**RMSSD [ms]**	59.8	61.4	59.3	67.6	55.2	58.4	56.7	52.9
	**pNN50 [%]**	17.3	8.4	10.9	8.0	10.7	10.6	8.3	8.0
**Freq.-domain HRV**	**HF [ms^2]**	600	600	600	1200	600	800	1300	900
	**LF [ms^2]**	1200	1000	900	3100	800	1200	2100	1200
	**HF$_{NU}$**	0.418	0.378	0.457	0.388	0.467	0.502	0.516	0.457
	**LF$_{NU}$**	0.583	0.622	0.543	0.612	0.534	0.498	0.484	0.543
	**LF/HF**	1.985	2.454	1.796	2.424	1.594	1.388	1.291	1.832
	**HF$_{NU}$ var**	0.022	0.029	0.030*	0.022‡	0.023	0.030	0.027	0.024
**CRC**	**HFc**	0.785	0.756	0.737*	**0.638**	0.792	0.792	0.781	**0.735**
	**LFc**	0.426	0.424	0.433	0.475	0.423	0.464	0.428	0.475
	**HFc var**	0.012	0.018	0.027*	**0.057**	0.010	0.011	0.010	**0.030**

HRV and CRC parameter values are calculated overnight from RR intervals and nasal pressure. Data are presented as median for each quartile and the statistical significance of the difference between quartiles was assessed with the Mann-Whitney U-test. Abbreviations: HRV = Heart rate variability, CRC = Cardiorespiratory coupling measured as the spectral coherence between RR intervals and nasal pressure, PVT = Psychomotor vigilance task, BMI = Body mass index, TST = Total sleep time, REM = Rapid eye movement, AHI = Apnea-hypopnea index, ODI = Oxygen desaturation index, T90 = Time spent in oxygen desaturation < 90%, DesSev = Desaturation severity, RT = Reaction time, ESS = Epworth Sleepiness Scale, SDNN = standard deviation of NN intervals (artifacts corrected), RMSSD = Root mean square of successive differences, pNN50 = Proportion of adjacent NN intervals differing more than 50 ms, HF = High-frequency band (0.15-0.4 Hz), LF = Low-frequency band (0.04-0.15 Hz), HF$_{NU}$ = Normalized power in the HF band (HF/(HF+LF)), LF$_{NU}$ = Normalized power in the LF band (LF/(HF+LF)), LF/HF = Ratio of low-frequency to high-frequency band power, var = variance, HFc = Cross spectral coherence in HF band, LFc = Cross spectral coherence in LF band.

* = statistically significant difference compared to Q_1 ($p < 0.008$). † = statistically significant difference compared to Q_2 ($p < 0.008$), ‡ = statistically significant difference compared to Q_3 ($p < 0.008$). Bolded values denote statistically significant differences compared to all other quartiles ($p < 0.008$).

Table S5. HRV, CRC, and performance in PVT in groups based on HF_{NU}.

		Men				Women			
		Q_1	Q_2	Q_3	Q_4	Q_1	Q_2	Q_3	Q_4
**N of patients**		72	72	72	72	64	65	65	64
**Age [y]**		56.1	47.9	47.7	55.6	49.6	48.0	51.3	54.9
**BMI [kg/m^2]**		32.2	31.8	30.5	33.8*‡	36.7	34.8	35.9	36.7
**TST [min]**		424.0	431.3	422.3	419.5	419.5	426.5	434.0	412.5
**Sleep Stages**	**N1 [min]**	55.8	43.3	42.0	37.0*	24.8	25.0	29.5	25.3
	**N2 [min]**	164.3	159.8	164.3	160.3	160.5	165.5	165.0	162.0
	**N3 [min]**	33.3	45.8	52.3	65.5*	80.3	80.5	69.0	81.5
	**REM [min]**	50.0	64.0	56.3	49.5†	61.3	64.0	66	63
**AHI [events/h]**		18.2	18.1	17.0	18.3	9.1	8.6	10.9	9.5
**ODI [events/h]**		11.8	13.6	11.6	17.1	7.4	4.9	5.1	7.5
**T90 [min]**		6.9	4.9	2.0	11.7‡	2.3	1.2	1.1	3.4
**DesSev [%]**		0.324	0.372	0.249	0.499	0.187	0.086	0.129	0.168
**PVT**	**Median RT [ms]**	352	372.5	363	361	410.5	400	388	396
	**Lapses**	7	11	11.5	8.5	25	19	14	19
**ESS**		10	11	10	9.5	9	11	12	10.5
**Time-domain HRV**	**Mean NN [ms]**	936.0	875.9	951.4	923.1	834.8	883.2	888.4*	888.6*
	**SDNN [ms]**	96.2	91.5	95.1	92.3	82.3	87.4	80.4	80.4
	**RMSSD [ms]**	53.8	51.2	59.7	**79.7**	47.4	55.2	51.5	68.7*‡
	**pNN50 [%]**	8.1	8.0	14.2*	17.3*†	**3.7**	13.9*	11.3*	15.4*
**Freq.-domain HRV**	**HF [ms^2]**	500	400	700	1400†	900	800	700	1200
	**LF [ms^2]**	1700	800	1100	1200	1600	2500	700	900
	**HF_{NU}**	-	-	-	-	-	-	-	-
	**LF_{NU}**	**0.749**	**0.639**	**0.545**	**0.386**	**0.696**	**0.547**	**0.456**	**0.319**
	**LF/HF**	**4.603**	**2.530**	**1.682**	**0.912**	**3.606**	**1.756**	**1.156**	**0.659**
	**HF_{NU} var**	**0.019**	0.026*	0.029*	0.029*	0.022	0.028	0.029*	0.025
**CRC**	**HFcoh**	0.736	0.735	0.754	0.754	0.753	0.788	0.786*	0.783*
	**LFcoh**	0.478	0.425	0.430	0.428	0.423	0.427	0.464	0.428
	**HFcoh var**	0.029	0.028	0.021	0.022	0.019	0.011	0.010*	0.015

HRV and CRC parameter values are calculated overnight from RR intervals and nasal pressure. Data are presented as median for each quartile and the statistical significance of the difference between quartiles was assessed with the Mann-Whitney U-test. Abbreviations: HRV = Heart rate variability, CRC = Cardiorespiratory coupling measured as the spectral coherence between RR intervals and nasal pressure, PVT = Psychomotor vigilance task, BMI = Body mass index, TST = Total sleep time, REM = Rapid eye movement, AHI = Apnea-hypopnea index, ODI = Oxygen desaturation index, T90 = Time spent in oxygen desaturation < 90%, DesSev = Desaturation severity, RT = Reaction time, ESS = Epworth Sleepiness Scale, SDNN = standard deviation of NN intervals (artifacts corrected), RMSSD = Root mean square of successive differences, pNN50 = Proportion of adjacent NN intervals differing more than 50 ms, HF = High-frequency band (0.15-0.4 Hz), LF = Low-frequency band (0.04-0.15 Hz), HF_{NU} = Normalized power in the HF band (HF/(HF+LF)), LF_{NU} = Normalized power in the LF band (LF/(HF+LF)), LF/HF = Ratio of low-frequency to high-frequency band power, var = variance, HFc = Cross spectral coherence in HF band, LFc = Cross spectral coherence in LF band.

* = statistically significant difference compared to Q_1 ($p < 0.008$). † = statistically significant difference compared to Q_2 ($p < 0.008$), ‡ = statistically significant difference compared to Q_3 ($p < 0.008$). Bolded values denote statistically significant differences compared to all other quartiles (p <-0.008).
